# The New York Homœopathic Union

**Published:** 1892-01

**Authors:** L. M. Stanton

**Affiliations:** Secretary; 71 West 88th St., New York


### THE NEW YORK HOMŒOPATHIC UNION.

### MINUTES OF FIRST FALL MEETING.

The first meeting of the season was held October 15th, at Dr. Carleton's office.

Dr. Fincke was unanimously re-elected President and occupied the chair.

Dr. Fincke said he had recently had the pleasure of seeing Dr. P. P. Wells, and that he—Dr. Wells—was in much better health, having found the remedy homœopathic to his condition.

Sections 117–121 of the *Organon* were read.

Dr. Fincke said that in section 117 it is made clear that an idiosyncrasy is simply an extreme sensibility on the part of an individual toward the action of a certain substance. The action of a medicine never varies, but individuals respond differently to different parts of its action. The symptoms from which these sensitive persons suffer are to be considered part of the proving of that particular substance to which they are sensitive.

Again, a person will experience certain symptoms from a low potency, another set of symptoms from a higher potency, and a third group from a third change in potency, and so on, hence many provings only show the individuality of a remedy, and from many provings only do we get a finished harmonious picture.

In speaking of the idiosyncrasies, Dr. Walter M. James, of Philadelphia, reported a case of a weakly tuberculous child, having a cough always brought on by sleeping on a feather-bed or on laying the head on a feather pillow.

Dr. Morgan said when a boy he could never eat hickory-nuts without suffering from a crop of boils in consequence. It was suggested that in potency these nuts should be useful in the treatment of boils.

Dr. Carleton knew of a case of a child in collapse, with a craving for cheese; some was given to the child and marked improvement followed. Likewise in a case of croup there was improvement in the child's condition after eating cheese.

Dr. Carleton said on being called to see a lady he was requested by her not to give Ipecac; she had taken it in different potencies, but it always made her deathly ill. Her symptoms indicated Ipecac, but Dr. Carleton said he feared to give it and selected another remedy.

Dr. Fincke thought that some persons were so susceptible to certain medicines that it made no difference how high you might give a particular medicine, the person could always detect it.

Following the reading of section 119, the question again came up of giving deep-acting remedies to cases that were incurable. Dr. Howard said he remembered Dr. Bayard having a case in the last stage of consumption where Sulphur was indicated. Dr. Bayard gave it and great improvement followed.

Dr. James told of a case of Dr. H. N. Guernsey's—one of typhoid fever—where Dr. Lippe was called in consultation. Merc. was clearly indicated, but Dr. Lippe advised strongly against giving it. Later in the case the symptoms still pointed to Merc. Dr. Guernsey gave it and death followed very shortly.

Dr. Carleton was reminded of a case of consumption which called for Sulphur, but not wishing to give it, selected Sang. as the remedy that came nearest to it in this case. The man improved decidedly under Sang., and was zigzagged back to health without the use of Sulph.

Dr. Fincke thought that at present we could not decide this question ; a good deal more experience only could do that.

Hinging on the above, a discussion arose as to what was an incurable case. Dr. Dyer thought that very often we could tell whether a case was incurable. The majority of the members, however, seemed to think that this could not be decided. Many so-called incurable cases come to us from allopathic hands and are cured, and even among ourselves a case which seems hopeless will recover under the well-selected remedy.

Dr. Morgan said he had a case of Bright's disease, the relative of an old-school physician, who was considered to be beyond cure. Dr. Morgan observed on one occasion that the man showed great disgust and was made sick by the smell of some eggs that were brought to him. Colchicum was given and the man recovered. At that time no kidney symptoms were found in the provings of Colch. Subsequent provings, however, show that it has an action on these organs. But the characteristic symptoms, nauseated by the smell of food, led Dr. Morgan to the remedy that cured the case.

Dr. Howard told of a case of uræmic convulsions with nausea, cold, clammy sweat, coldness of legs, twitching of the muscles, head rolling from side to side, collapse. Dr. Howard gave Bell. 9th, and cured.

Dr. Fincke told of a case of a dying man with the death-rattle, large ecchymoses covering the chest, where euthanasia followed a dose of a high potency of Camph.

Section 120 was read and the meeting adjourned.

At the close Dr. Arschaganni said that he had been advised by an eminent French physician to come to America to study Homœopathy, that he would remain with us another year and then return to practice in Constantinople. He said Homœopathy was spreading in the East, and that there were many good homœopathic physicians in Russia.

L. M. Stanton,
71 West 88th St., New York. *Secretary.*

